# Cutaneous Rosai-Dorfman Disease Misdiagnosed as Granulomatous Dermatitis: A Diagnostic Pitfall

**DOI:** 10.7759/cureus.105191

**Published:** 2026-03-13

**Authors:** Sri Naidnur, Jamie Hedrick, Smaran Marupudi, Kara Asbury, Kelly Maedo, Bryan Gammon, Rick Lin

**Affiliations:** 1 Dermatology, Oasis Dermatology Group, McAllen, USA; 2 Dermatology, University of Texas Rio Grande Valley School of Medicine, McAllen, USA; 3 Dermatology, HCA Healthcare Corpus Christi Medical Center-Bay Area Program, McAllen, USA; 4 Dermatopathology, Sagis Diagnostics, Houston, USA

**Keywords:** cd163, cd68, clinicopathologic correlation, crdd, cutaneous rosai-dorfman disease, diagnostic pitfall, emperipolesis, granulomatous dermatitis, non-langerhans cell histiocytosis, s100

## Abstract

Cutaneous Rosai-Dorfman disease (CRDD) is an uncommon non-Langerhans cell histiocytosis that may occur without systemic involvement and lacks specific clinical features. We report a 17-year-old female with a slowly enlarging chest lesion initially diagnosed as granulomatous dermatitis on a 4-mm punch biopsy. The lesion was treated as an inflammatory process without improvement. Complete excision revealed a multinodular dermal and subcutaneous infiltrate of large histiocytes demonstrating emperipolesis, confirming CRDD. Systemic evaluation was negative. The initial limited biopsy did not capture defining features, delaying diagnosis.

Persistent nodular lesions that fail to respond to therapy should prompt reconsideration and more representative tissue sampling. We have also tabulated selected reported cases, which further demonstrate that CRDD is frequently misdiagnosed in routine clinical practice due to nonspecific morphology and limited biopsy sampling.

## Introduction

Rosai-Dorfman disease (RDD) is a rare non-Langerhans cell histiocytosis with an estimated prevalence of approximately 5 cases per 1,000,000 individuals (≈1 in 200,000) [1]. It is classically associated with massive cervical lymphadenopathy; however, the disease may be limited to the skin without systemic involvement [2,3]. Cutaneous manifestations occur in approximately 10% of patients with systemic disease, and purely cutaneous involvement alone is rarer [2-4].

Cutaneous RDD (CRDD) most commonly affects middle-aged adults, demonstrates a slight female predominance, and frequently involves the face and trunk, although presentations in adolescence are less typical [3,4]. CRDD may present as papules, nodules, or plaques resembling inflammatory, infectious, or malignant processes, which can delay recognition [3].

Histopathologically, CRDD is characterized by large histiocytes with abundant pale cytoplasm demonstrating emperipolesis (the presence of intact lymphocytes or plasma cells within the cytoplasm of histiocytes, which are tissue macrophages) within a mixed inflammatory infiltrate [2,4]. When these defining features are not captured on limited sampling, the diagnosis may be obscured or interpreted as nonspecific granulomatous dermatitis, as in this case, highlighting an important diagnostic pitfall.

## Case presentation

**Figure 1:**
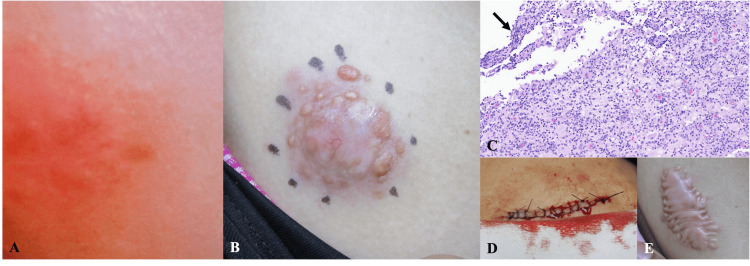
Clinical and histopathologic images A. Erythematous patch with subtle clustered papules approximately one year before presentation B. Flesh-colored to pink papules coalescing into a plaque overlying a firm subcutaneous nodule; peripheral ink markings outline the lesion margins C. H&E stain (10×) demonstrating superficial suppurative and granulomatous inflammation with overlying purulent crust (arrow), suggestive of a ruptured follicle or cyst D. Immediate post-excision view with linear closure E. Nine-month follow-up showing a well-healed surgical site with keloid formation H&E, hematoxylin and eosin.

A 17-year-old female presented with a one-year history of a slowly enlarging lesion on the right anterior chest. She shared a prior photograph showing the lesion at an earlier stage, when it appeared as an ill-defined erythematous patch with subtle papular change and no appreciable nodularity (Figure 1A). Over the course of one year, the lesion evolved into clustered flesh-colored to pink papules coalescing into plaques overlying a palpable subcutaneous nodule (Figure 1B). Clinically, the lesion measured approximately 3.0 × 2.5 cm. The lesion was asymptomatic, and the patient denied preceding trauma, fevers, weight loss, or other systemic symptoms. No palpable lymphadenopathy was identified on examination.

The clinical differential diagnosis included epidermal inclusion cyst, keloid, dermatofibrosarcoma protuberans, Merkel cell carcinoma, and molluscum contagiosum. An initial 4-mm punch biopsy demonstrated superficial suppurative and granulomatous inflammation with overlying purulent crust, findings interpreted as nonspecific granulomatous dermatitis likely secondary to a ruptured follicle or cyst (Figure 1C). The patient was treated with topical benzoyl peroxide, clindamycin, and oral doxycycline for approximately five months without improvement. Given the persistence of the lesion, surgical excision was pursued (Figure 1D). The excision site healed with keloid formation (Figure 1E).

**Figure 2:**
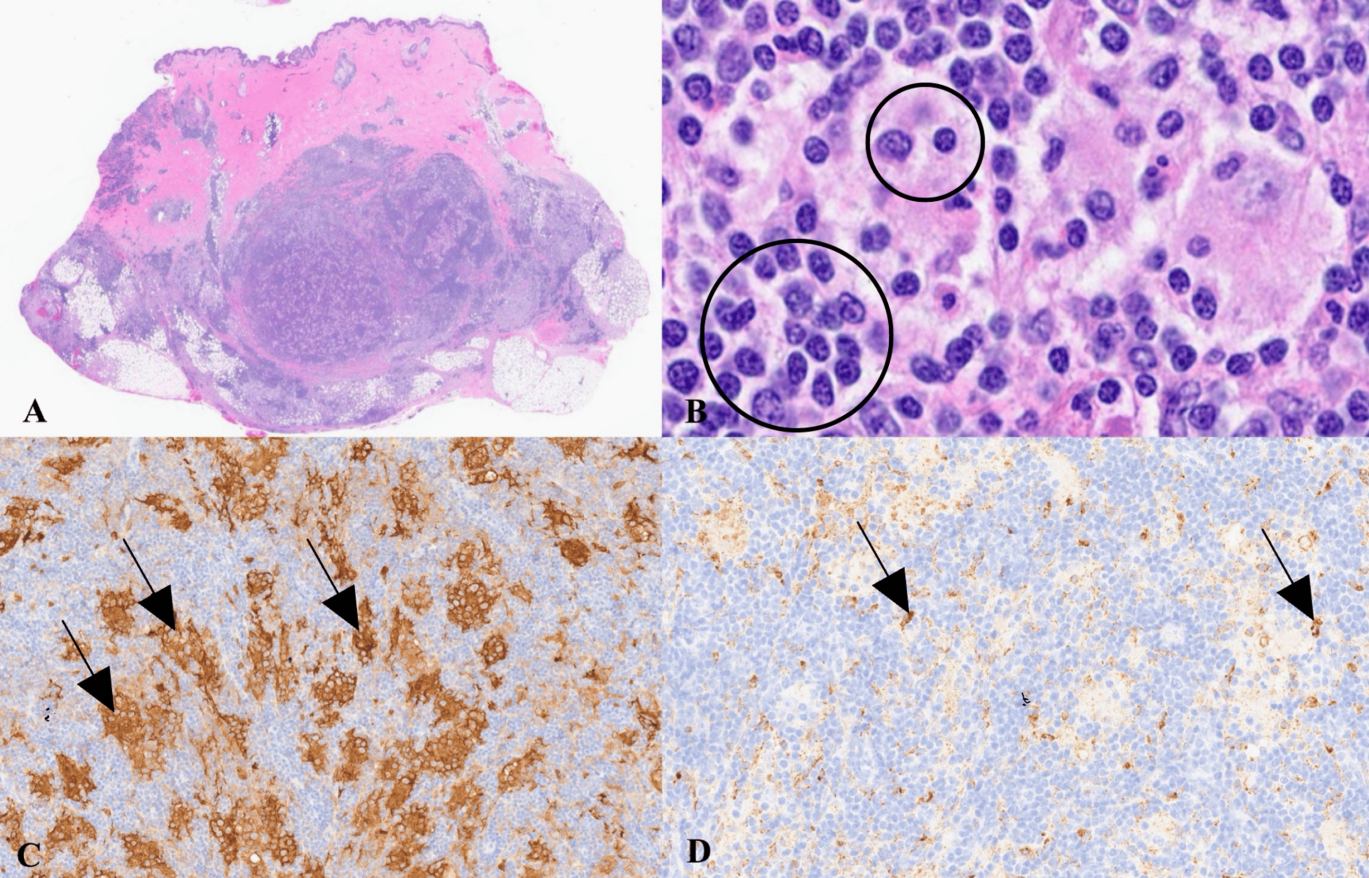
Histopathologic and immunohistochemical features of CRDD A. Low-power H&E staining demonstrating a well-circumscribed, multinodular dermal infiltrate extending into the subcutis (original magnification ×20). B. High-power H&E staining showing large histiocytes with prominent emperipolesis (circled), characterized by intact lymphocytes within the cytoplasm of histiocytes (original magnification ×400). C. S100 IHC demonstrating strong nuclear and cytoplasmic positivity in large histiocytes (arrows) within the dermal infiltrate (original magnification ×200). D. CD68 IHC showing granular cytoplasmic positivity in histiocytes (arrows) within the dermal infiltrate (original magnification ×200). CD, cluster of differentiation; H&E, hematoxylin and eosin; IHC, immunohistochemistry.

Gross examination revealed a well-circumscribed subcutaneous mass measuring approximately 4.0 × 3.5 cm. On hematoxylin and eosin staining, sections demonstrated a dense dermal and subcutaneous infiltrate composed of large histiocytes with abundant pale cytoplasm exhibiting emperipolesis within a mixed inflammatory background of lymphocytes and plasma cells (Figures 2A, 2B). Immunohistochemical studies demonstrated positivity for S100 (Figure 2C), CD68 (Figure 2D), and CD163, supporting a diagnosis of CRDD.

The patient was referred to pediatric hematology/oncology for systemic workup. Contrast-enhanced chest computed tomography was unremarkable. Whole-body fluorine-18-deoxyglucose positron emission tomography demonstrated no definitive pathologic nodal or extranodal involvement. A cancer mutation panel performed on the excised tissue did not identify pathogenic alterations in mitogen-activated protein kinase (MAPK) pathway genes. At the nine-month follow-up post-excision, the patient remained recurrence-free and without systemic involvement.

## Discussion

Clinically, CRDD lacks specific features and may resemble inflammatory, infectious, or neoplastic processes, contributing to diagnostic delay [3]. Histologically, it is defined by large histiocytes with abundant pale cytoplasm demonstrating emperipolesis within a mixed inflammatory infiltrate [2,4]. Lesional histiocytes express S100 and macrophage-associated markers, such as CD68 and CD163, and lack CD1a, a Langerhans cell marker, supporting non-Langerhans cell differentiation [4].

Although historically considered a reactive condition, RDD is now recognized as biologically heterogeneous. Activating mutations involving the MAPK signaling pathway have been identified in a subset of cases, supporting clonal histiocytic proliferation in those patients [5]. However, not all cases harbor detectable mutations, and the clinical course is often indolent [4,5]. In our patient, a cancer mutation panel did not identify pathogenic MAPK pathway alterations.

CRDD has been misdiagnosed in a variety of clinical contexts. Selected previously reported cases (Table 1) highlight recurring pitfalls at both the clinical and histopathologic levels. Contributing factors include anatomic location, epidemiologic context, nonspecific morphology, limited sampling, and under-recognition of characteristic features [6-15]. A focused literature search was performed using the terms “cutaneous Rosai-Dorfman disease”, “misdiagnosis”, and “initial diagnosis.”

**Table 1:** Selected reported cases of cutaneous Rosai-Dorfman disease initially misdiagnosed CD, cluster of differentiation; CRDD, cutaneous Rosai-Dorfman disease; H&E, hematoxylin and eosin; IHC, immunohistochemistry; +, positive; −, negative.

Author (year)	Age/sex	Location	Initial diagnosis	Diagnostic clarification
Stefanato et al. (2002) [6]	55/F	Back and lower extremities	Vasculitis	Clinical misdiagnosis; H&E confirmed CRDD
Asawabenjang et al. (2013) [7]	66/M	Right flank	Chronic eczema	H&E demonstrated emperipolesis; IHC (S100+, CD1a−) supported CRDD
Kaskas et al. (2015) [8]	53/F	Scalp (vertex)	Granulation tissue with inflammation	Deep shave biopsy interpreted as granulation tissue; subsequent excisional biopsy showed emperipolesis on H&E and S100+ histiocytes
Mizuta et al. (2021) [9]	51/M	Right temporal scalp	Angiosarcoma	Re-evaluation demonstrated emperipolesis on H&E; IHC (S100+, CD68+, CD1a−, CD34−, ERG−) supported CRDD
Arya et al. (2020) [10]	27/F	Malar and right axillary regions	Acne	H&E demonstrated emperipolesis; IHC (S100+, CD68+) supported CRDD
St. Claire et al. (2023) [11]	65/M	Right lower abdomen	Granulation tissue with inflammation	Punch biopsy nondiagnostic; excisional biopsy with H&E and IHC (S100+, CD163+, CD1a−) supported CRDD
Sheetz et al. (2025) [12]	53/F	Right knee to shin	Atypical polyclonal lymphohistiocytic infiltrate	Repeat biopsy demonstrated emperipolesis on H&E; IHC (S100+, CD68+, CD1a−) supported CRDD
Bogomolets et al. (2025) [13]	23/F	Right cheek and left arm	Acne conglobata	Clinical misdiagnosis; diagnosis established after six biopsies demonstrating emperipolesis on H&E and S100 positivity
Dsouza et al. (2026) [14]	18/M	Nasal bridge and right forearm	Granulomatous dermatitis	Additional/serial sectioning demonstrated emperipolesis on H&E; findings supported CRDD
Wang et al. (2026) [15]	64/F	Right cheek	Sporotrichosis	Re-review of original biopsy demonstrated emperipolesis on H&E; IHC (S100+, CD68+, CD1a−, CD207−) supported CRDD

Our case highlights a particularly relevant pitfall: granulomatous dermatitis represents a reaction pattern rather than a definitive diagnosis and may obscure CRDD on limited sampling. When a lesion persists or evolves despite appropriate therapy for the presumed diagnosis, clinicopathologic correlation is essential. Reconsideration of the differential diagnosis and procurement of more representative tissue may prevent diagnostic delay and unnecessary treatment, including prolonged antibiotic exposure. 

Given the potential for recurrence, periodic clinical follow-up every 6-12 months was recommended.

## Conclusions

Cutaneous RDD can mimic inflammatory dermatoses on limited biopsy, making adequate tissue sampling essential for accurate diagnosis. Awareness of this diagnostic pitfall may help reduce diagnostic delays and avoid prolonged empiric therapy.
